# Radiation Protection Practices Among Medical Students at Two United Kingdom Medical Schools

**DOI:** 10.7759/cureus.117065

**Published:** 2026-09-28

**Authors:** Areen Tawil, Aysha Al-Bakri

**Affiliations:** 1 Internal Medicine, New Cross Hospital, Wolverhampton, GBR

**Keywords:** ionising radiation, medical education, occupational radiation exposure, protective equipment, radiation safety

## Abstract

Background

Orthopaedic surgery frequently involves the use of intraoperative fluoroscopy, exposing operating theatre personnel to ionising radiation. Medical students often attend orthopaedic procedures during their clinical training, yet their education on radiation safety and access to appropriate protective equipment remain poorly understood. This study aimed to evaluate radiation safety among medical students during surgical placements, focusing on educational exposure, practical radiation protection practices, and perceived safety.

Methodology

A multicentre, cross-sectional, questionnaire study was conducted mainly among UK medical students from two medical schools in clinical years (n = 70). A structured nine-item questionnaire was distributed via Google Forms over a 10-day period in December 2023. Participants were recruited through university WhatsApp groups. Participation was voluntary and anonymous, and completion of the questionnaire was taken as implied informed consent.

Results

A total of 70 medical students completed the survey (44% female, 30% male, 26% preferred not to say). Only 50% reported wearing an appropriately fitting lead apron, while 41% reported inadequate fit. Consistent use of a thyroid shield was reported by 41% of respondents, whereas 49% reported not wearing one. Formal radiation safety teaching before orthopaedic theatre attendance had been received by only 16% of participants. One-third (33%) reported feeling unsafe in operating theatres where ionising radiation was used.

Conclusions

The medical students surveyed who attended the theatre where ionising radiation was used demonstrated important gaps in radiation safety education and inconsistent use of appropriate protective equipment. Introducing structured radiation safety teaching before theatre exposure and ensuring access to correctly fitted protective equipment may improve student safety, confidence, and preparedness in the operating theatre.

## Introduction

Orthopaedic surgery relies heavily on ionising radiation (IR) for intraoperative imaging and procedural guidance. Since its introduction in 1895, radiographic imaging has become integral to orthopaedic practice [1]. Despite its clinical use, occupational exposure to IR carries well-established risks.

Concerns regarding the long-term health effects of radiation exposure have been raised. Female orthopaedic surgeons have been reported to have a 2.9-fold increased risk of breast cancer compared with the general population [2]. This highlights the importance of effective radiation protection measures within the surgical environment.

Medical students frequently attend orthopaedic theatres during clinical placements, where they may be exposed to IR with limited formal training in radiation safety. As future clinicians, it is essential to ensure they are adequately protected and educated.

IR is widely used in orthopaedic surgery, particularly through fluoroscopic imaging for fracture fixation and intraoperative guidance [3]. Despite IR being essential for clinical practice, exposure is associated with cumulative stochastic risks, including malignancy and cataract information [4]. In the United Kingdom, the safe use of IR is governed by the Ionising Radiations Regulations 2017, which outline employer responsibilities for radiation protection, including training, risk assessment, and provision of appropriate protective equipment [5]. The principle of optimisation of radiation protection outlined in the 2007 recommendations of the International Commission on Radiological Protection (ICRP) is also fundamental in minimising occupational exposure [6]. It is a foundational concept which emphasises that exposure to IR should be minimised as much as possible, considering economic and social factors.

Research has demonstrated that orthopaedic surgeons are at increased risk due to fluoroscopically guided procedures. Increased radiation exposure to the hands, thyroid, and eyes is common during orthopaedic procedures such as intramedullary nailing and dynamic hip screw fixation [7]. However, there is still inconsistent use of personal protective equipment (PPE), including thyroid shields and lead glasses, even if available [8]. Poor compliance is likely attributed to lack of education about radiation risks, lack of formal training and time constraints [9,10]. Educational interventions can significantly improve understanding of radiation doses, and hence increase PPE use and optimise positioning during fluoroscopic procedures [11].

Furthermore, there remains a lack of focus regarding educating medical students. As temporary members of the operating theatre, they often lack formal induction and access to appropriate PPE. Hence, their understanding largely depends on inconsistent informal teaching. Surveys assessing medical student knowledge showed inadequate understanding of protective strategies and low confidence in applying these in clinical practice [12]. A cohort study assessed medical students’ knowledge after a formal lecture, which showed an increase of 31% [13].

Existing literature highlights three key issues, including insufficient formal teaching, variability in safe practice, and a lack of focus on undergraduate populations. This study aims to evaluate radiation safety among medical students attending theatres, with specific objectives to assess educational exposure to radiation safety, practical radiation protection practices, including PPE use and positioning, and students’ perceptions of safety during theatre attendance.

## Materials and methods

A quantitative, cross-sectional, questionnaire-based study was conducted among UK medical students undertaking clinical training from mainly two UK medical schools. Recruitment was primarily undertaken through medical student networks at the University of Plymouth and the University of Manchester. Data were collected over a 10-day period in December 2023 using an anonymous online questionnaire administered through Google Forms. The study aimed to assess medical students’ exposure to radiation safety education, use of PPE, education surrounding radiation protection practices, and perceptions of safety when attending operating theatres in which IR was used.

Participants were eligible for inclusion if they were medical students enrolled at a UK medical school and were undertaking clinical training at the time of the survey. Students were required to have experience of, or exposure to, clinical environments in which IR could be used, particularly operating theatres. Participation was voluntary.

The questionnaire was distributed using convenience sampling through university medical student WhatsApp group chats, primarily at the University of Plymouth and the University of Manchester. Students from other UK medical schools who received the survey link and met the eligibility criteria were also eligible for inclusion.

A total of 70 medical students completed the questionnaire and were included in the analysis. Of these, 56% (39) were from the University of Plymouth, 40% (28) were from the University of Manchester, and 4% (3) were from other UK medical schools (Cambridge, Imperial College London, and University of Lancashire).

The total number of students who received the survey invitation could not be reliably established because recruitment occurred through WhatsApp group chats and the survey link could be accessed and shared within student networks. Consequently, an accurate denominator for the invited population was unavailable, and a formal response rate could not be calculated.

Data were collected using a structured nine-item questionnaire developed specifically for this study by the authors, Areen Tawil and Aysha Al-Bakri, and administered electronically using Google Forms. The questionnaire was designed collaboratively by both authors in accordance with the study objectives and focused on radiation protection issues relevant to medical students attending clinical environments where IR may be used. The full questionnaire is included in the Appendices.

The questionnaire was not adapted directly from a previously validated instrument and was therefore treated as a study-specific survey rather than a validated psychometric scale. The questionnaire was reviewed by clinicians with relevant experience before distribution to assess the relevance and clarity of the items.

The nine questionnaire items covered the following principal domains: (1) Participant characteristics, including sex and university. (2) PPE, including perceived appropriateness of lead apron fit and use of thyroid protection. (3) Radiation safety behaviour and guidance, including whether students were reminded where to stand to minimise radiation exposure. (4) Radiation safety education, including whether formal radiation safety teaching had been received before exposure to IR in theatre. (5) Perceived safety, including whether participants felt safe when attending theatres where IR was being used. Questions used categorical response formats appropriate to each item, including binary responses (e.g. yes/no) and multiple-choice categorical responses. There was one question titled additional comments, which was a free-text question. As the questionnaire assessed several distinct aspects of radiation safety rather than a single underlying construct, no overall questionnaire score was calculated. Each item was analysed independently.

For the purposes of this study, formal radiation safety training was defined as participants reporting that they had received dedicated radiation safety teaching before exposure to IR in the theatre environment. This definition was developed specifically for the purposes of this study, as there is currently no standardised guidance specifying the radiation safety information or formal training that medical students should receive before attending clinical environments where IR may be used. Appropriate PPE practice was assessed separately according to reported lead apron fit and thyroid shield use rather than being combined into a composite score.

Data collection took place over a 10-day period in December 2023. Participants accessed the questionnaire through an electronic link to Google Forms. Participation was voluntary, and questionnaire responses were anonymised to maintain confidentiality. No personally identifiable information was collected. Participants were informed about the purpose of the questionnaire, and completion and submission of the survey were taken to indicate informed consent. No formal prior sample size calculation was performed because this was an exploratory student-led survey using convenience sampling. As the study was primarily descriptive, categorical variables were summarised using frequencies and percentages. This included participant sex, university affiliation, reported lead apron fit, thyroid shield use, receipt of radiation safety teaching, reminders regarding positioning to minimise radiation exposure, and perceived safety in theatres where IR was present.

The project was conducted as a student-led service evaluation and therefore did not undergo formal research ethics committee review. Participation was voluntary and anonymous, no personally identifiable information was collected, and completion of the questionnaire was taken as implied consent. The service evaluation classification should be considered when interpreting the governance and ethical oversight of the study.

## Results

A total of 70 medical students completed the questionnaire. Sex was reported by 52 participants. Overall, 31/70 (44%) of participants were female, 21/70 (30%) were male, and 18/70 (26%) preferred not to disclose their sex.

Following standardisation of the free-text responses for medical school affiliation, the majority of participants were associated with the University of Plymouth or the University of Manchester. Overall, 39 (56%) participants were from the University of Plymouth, 28 (40%) were from the University of Manchester, and 3/70 (4%) were from other UK medical schools. Recruitment was primarily conducted through medical student WhatsApp networks associated with Plymouth and Manchester. However, as the survey link could be shared within wider student networks, three eligible responses were received from students outside the two principal institutions and were retained in the analysis. Participant characteristics are summarised in Table 1.

**Table 1:** Participant characteristics (N = 70). The year of study was not collected in the questionnaire and therefore could not be reported.

Characteristic	n/N	%
Sex		
Female	31/70	44
Male	21/70	30
Prefer not to disclose	18/70	26
Medical school		
University of Plymouth	39/70	55
University of Manchester	28/70	40
Other UK medical schools	3/70	4

Previous exposure to ionising radiation

All 70/70 (100%) participants reported having previously been present during at least one clinical procedure involving IR. Four (6%) participants reported attending one such procedure, five (7%) reported attending two, 10 (14%) reported attending three, and eight (12%) reported attending four. The largest group, 43 (61%), reported having attended five or more procedures involving IR.

Personal protective equipment

Only half of participants, 35 (50.0%), responded that their lead apron adequately covered their breast and axillary regions. Overall, 29 (41%) participants reported that it did not. The remaining 6/70 (9%) provided qualified responses indicating partial, inconsistent, or uncertain protection. Several of these responses specifically indicated that the breast region was covered, but the axillary region was not, while another participant reported variation in protection between different clinical settings.

Thyroid shield use was similarly inconsistent. Overall, 29 (41%) participants reported wearing a thyroid shield during procedures involving IR, while 34 (49%) reported that they did not. A further seven (10%) described intermittent or conditional use, including wearing a thyroid shield only occasionally, when available, or after specifically requesting one.

Radiation safety guidance and education

Overall, 37 (53%) participants reported being reminded where to stand to reduce their radiation exposure, while 30 (43%) reported that they were not given such guidance. The remaining 3/70 (4%) indicated that they were reminded only sometimes.

Formal preparation before theatre exposure was uncommon. Only 11 (16%) participants reported receiving an introduction to radiation safety before theatre attendance, whereas 56 (80%) reported receiving no such introduction. Three participants (4%) gave uncertain or conditional responses, including “sometimes” and “not sure.”

Perceived safety in theatre

Overall, 66 participants answered the question regarding whether they felt safe in theatres where IR was present. Of these, 44 (67%) reported feeling safe, whereas 22 (33%) reported feeling unsafe. Four participants did not answer this question. The complete questionnaire findings are summarised in Table 2.

**Table 2:** Questionnaire responses relating to radiation exposure and safety. Data are presented as n/N (%). Percentages are based on the number of valid responses to each question. Free-text responses provided within categorical questions were reviewed and grouped into descriptive categories while preserving their original meaning.

Questionnaire item	Response	n/N	%
Previous procedures involving ionising radiation	1 procedure	4/70	6
	2 procedures	5/70	7
	3 procedures	10/70	14
	4 procedures	8/70	12
	≥5 procedures	43/70	61
Lead apron adequately covered breast and axillary regions	Yes	35/70	50
	No	29/70	41
	Partial/Variable/Other	6/70	9
Thyroid shield worn	Yes	29/70	41
	No	34/70	49
	Intermittent/Conditional use	7/70	10.0
Reminded where to stand to reduce radiation risk	Yes	37/70	53
	No	30/70	43
	Sometimes	3/70	4
Introduction to radiation safety before theatre attendance	Yes	11/70	16
	No	56/70	80
	Other/Uncertain	3/70	4
Felt safe in theatres where radiation was present	Yes	44/66	67
	No	22/66	33

Qualitative findings

In total, five (7%) participants provided additional free-text comments. Given the small number of responses, these comments were analysed using descriptive content analysis rather than formal thematic analysis. Each response was read and coded according to the radiation safety concern or experience described. Similar codes were grouped into descriptive categories, and responses could contribute to more than one category when multiple concerns were raised.

The most frequently identified category related to the availability, adequacy, fit, or anatomical coverage of PPE, which was raised by 3/5 of free-text respondents. Specific concerns included insufficient availability and sizing of lead aprons, inadequate coverage of the axillary regions, lack of thyroid protection, and absence of eye protection.

One participant described insufficient safety oversight during clinical placement, reporting prior awareness of radiation risks but a lack of active staff intervention to ensure appropriate protection. One participant raised a concern regarding radiation exposure associated with compulsory orthopaedic theatre attendance. These qualitative findings are summarised in Table 3.

**Table 3:** Descriptive categories identified from free-text comments (n = 5 respondents). Categories were descriptive and were not considered formal qualitative themes because of the small number of free-text responses. Categories were not necessarily mutually exclusive. PPE = personal protective equipment

Descriptive category	Examples of concerns reported	n/N
PPE availability, adequacy, fit or coverage	Insufficient lead aprons/sizes; inadequate axillary coverage; absence of thyroid or eye protection	3/5
Insufficient radiation safety oversight	Lack of active staff assurance that appropriate radiation-protection measures were followed	1/5
Clinical placement/Exposure concerns	Radiation exposure associated with compulsory orthopaedic theatre attendance	1/5

## Discussion

This study identifies important gaps in radiation safety training, education, and protective practices among UK medical students, mainly from two institutions, during theatre exposure. Despite the well-established use of IR in orthopaedic procedures, our findings suggest that formal education and consistent implementation of safety measures at undergraduate level remain suboptimal.

A key finding of this study is that one-third of respondents reported feeling unsafe in theatre environments where IR was used. This perception is clinically important, as perceived safety is closely linked to confidence, engagement in theatre-based learning, and willingness to participate in surgical specialties [14]. Feelings of insecurity may reflect inadequate preparation before clinical exposure, inconsistent supervision, or variability in the enforcement of radiation protection protocols within theatre environments.

Notably, only 16% of respondents reported receiving formal radiation safety teaching before theatre exposure. This finding suggests a significant gap in undergraduate medical curricula, particularly given that orthopaedic theatres are commonly among the first surgical environments in which students are exposed to fluoroscopy and intraoperative imaging. The lack of structured teaching may contribute to poor understanding of fundamental radiation safety principles, including dose minimisation, appropriate positioning, and correct use of shielding equipment.

In addition, our findings demonstrate suboptimal use of PPE. Almost half of respondents reported not using a thyroid shield consistently, and 41% reported that their lead apron did not fit appropriately. This is concerning, as inadequate shielding of radiosensitive organs such as the thyroid and breast tissue may increase cumulative occupational exposure over time [15]. The issue of poor fit is particularly relevant for medical students, who may have limited access to appropriately sized protective equipment compared with permanent theatre staff.

These findings are consistent with previous literature highlighting variability in radiation safety culture within surgical specialties. While most operating theatres provide protective equipment, previous studies have shown that availability does not necessarily translate into consistent use, often due to lack of training, time pressures, or insufficient reinforcement from senior staff [16]. Our findings extend this concern to medical students, who may be particularly vulnerable due to their limited clinical experience and hierarchical position within the theatre environment.

Furthermore, the qualitative feedback in this study highlights additional practical concerns, including insufficient availability of appropriately sized lead aprons and lack of eye protection. Although less commonly discussed in undergraduate settings, ocular exposure to scatter radiation has been recognised as a potential occupational risk in interventional specialties [17]. The absence of eye protection provision suggests that radiation safety protocols may not be fully standardised for student-level exposure.

It is also notable that 43% of respondents reported not being reminded where to stand during radiation exposure. Proper positioning is a simple but highly effective method of reducing scatter radiation dose; therefore, this finding may reflect missed opportunities for informal teaching during clinical placements [18]. Reinforcement of safe positioning by theatre staff could represent a low-cost, high-impact intervention to improve safety awareness.

Importantly, despite these deficiencies, two-thirds of respondents reported feeling safe in theatre environments. This discrepancy between perceived safety and objectively reported gaps in training and PPE use may indicate a lack of awareness regarding radiation risks rather than the true absence of risk. This highlights the importance of improving not only safety infrastructure but also risk awareness and education.

From a systems perspective, these findings suggest that radiation safety for medical students is inconsistently implemented across clinical placements. Variability between institutions, theatre teams, and supervising clinicians may contribute to unequal learning experiences. Standardisation of induction processes before orthopaedic placements, including mandatory radiation safety briefings, may help address this inconsistency.

Educational interventions have been shown in other contexts to significantly improve radiation safety knowledge and behaviour among trainees [19]. Incorporating structured teaching sessions, simulation-based learning, or mandatory e-learning modules may improve both knowledge retention and compliance with safety protocols. In addition, ensuring that appropriately fitted lead protection is readily available to students should be considered a basic requirement for safe clinical exposure.

This study has several limitations. The sample size was relatively small and drawn mainly from two UK medical schools, which may limit generalisability to other institutions. As the survey was developed specifically for this study, its measurement properties have not been formally established. While clinical review was undertaken during questionnaire development to ensure that the questions were clear and relevant, further validation would strengthen the reliability and reproducibility of future research using this survey. The use of convenience sampling and a study-specific questionnaire may also limit the exact reproducibility of the study across different institutions and student populations.

In addition, the use of self-reported questionnaire data introduces potential recall and response bias. Students may have over- or under-reported PPE use or safety behaviours. Participants were recruited through university WhatsApp groups, which may have introduced selection bias, as students who chose to participate may have had a greater pre-existing interest in radiation safety. Additional participant characteristics, including year of study and the number and type of orthopaedic procedures previously attended, were not collected. These factors may influence students’ exposure to radiation safety practices and their familiarity with protective measures, and their absence limits further analysis of potential differences within the study population. Finally, the cross-sectional design limits causal inference, and the study cannot determine whether lack of training directly resulted in unsafe practices. Although previous exposure to radiation safety teaching was recorded, the questionnaire did not define or examine the format and extent of this teaching. Consequently, variation in educational exposure between participants could not be explored.

Despite these limitations, this study provides useful insight into an underexplored area of undergraduate surgical education. To our knowledge, few studies have specifically assessed radiation safety education among medical students in orthopaedic theatre settings. The findings therefore contribute to a growing recognition that radiation safety education should begin at the undergraduate level rather than being reserved for postgraduate training.

Future research should aim to expand sample size across multiple institutions and explore differences in radiation safety practices between specialties and clinical environments. Interventional studies assessing the impact of structured radiation safety teaching on knowledge, behaviour, and confidence would also be valuable.

## Conclusions

This exploratory study identified potential gaps in radiation safety education and access to appropriate protective equipment among the medical students surveyed. Structured teaching, access to appropriately fitted protective equipment, and reinforcement of safe practices may help improve students’ preparedness for clinical environments where IR is used. However, the small convenience sample, drawn predominantly from two medical schools, limits the generalisability of these findings. Further research involving larger and more representative samples is needed to determine the extent of these findings and inform future approaches to undergraduate radiation safety education.

## Appendices

Study questionnaire

Student Questionnaire: Radiation Safety During Theatre Attendance

1) How many times have you been present in a procedure where ionising radiation was used (e.g. X-ray)?

0

1

2

3

4

5+

2) Areas that would be vulnerable to radiation exposure are the breast and axillary regions. Do you feel your lead apron was accurately fitted to cover these areas?

Yes

No

Other: ______________________________

3) Did you wear a thyroid shield during your time in theatre where radiation was used?

Yes

No

Other: ______________________________

4) Were you reminded where to stand to reduce your radiation risk?

Yes

No

Other: ______________________________

5) Were you given an introduction to radiation safety prior to your theatre attendance?

Yes

No

Other: ______________________________

6) Do you feel safe when in theatres where radiation is present?

Yes

No

7) Please state which medical school you attend:

8) What is your sex?

Male

Female

Prefer not to say

9) Additional comments:
